# Morphometric variations and nonmetric anatomical traits or anomalies of the primary molar teeth, plus the molars' size thresholds for sex identification

**DOI:** 10.1186/s12903-024-03908-4

**Published:** 2024-02-07

**Authors:** Fataneh Ghorbanyjavadpour, Kosar Jamali, Maryam Roayaei Ardakani, Vahid Rakhshan

**Affiliations:** 1 Department of Orthodontics, School of Dentistry, Ahvaz Jundishapur University of Medical Sciences, Ahvaz, Iran; 2 Dentist, School of Dentistry, Ahvaz Jundishapur University of Medical Sciences, Ahvaz, Iran; 3 Department of Pediatric dentistry, School of Dentistry, Ahvaz Jundishapur University of Medical Sciences, Ahvaz, Iran; 4 Department of Anatomy, Dental School, Azad University of Medical Sciences, Tehran, Iran

**Keywords:** Orthodontics, Oral pathology, Pediatric dentistry, Dental anatomy, Primary dentition, Epidemiology, Metric dental traits, Mesiodistal width, Buccolingual width, Nonmetric dental traits, Shape anomalies or traits, Sex identification, Sex dimorphism, Anthropology, Morphology, Morphometry, Forensic science

## Abstract

**Introduction:**

Morphological and morphometric features of the teeth are of interest to various clinical and academic dental and medical fields including prosthodontics, orthodontics, anatomy and anthropology, pathology, archeology, and forensic dentistry. These have been more or less researched in the case of the permanent dentition. However when it comes to the primary dentition, the literature is scarce and controversial. No study worldwide exists on the cutoff points (thresholds) for sex identification; no study exists on metric or nonmetric traits of deciduous teeth in Iranians. Hence, the aim of the study was to assess both the metric and nonmetric traits of primary molars, as well as their cut-off points for sex identification.

**Methods:**

In this epidemiological cross-sectional study, pretreatment casts of 110 children (51 boys and 59 girls) aged 6 to 12 years were collected. Maxillary and mandibular first and second primary molars were evaluated regarding their metric traits (mesiodistal and buccolingual widths) and 9 nonmetric traits (Accessory cusp on the upper D, Accessory cusp on the lower D, Fifth cusp on the upper E, Carabelli’s cusp on the upper E, Protostylid on the lower E, Fifth cusp on the lower E, Sixth cusp on the lower E, Tuberculum intermedium [metaconulid] on the lower E, and Deflecting wrinkle on the lower E). ROC curves were used to identify cut-off points for sex determination as well as the usefulness of metric measurements for this purpose. Data were analyzed using independent-samples and paired-samples t-tests, McNemar, Fisher, and chi-square tests, plus Pearson and Spearman correlation coefficients (α = 0.05).

**Results:**

All the primary molars’ coronal dimensions (both mesiodistal and buccolingual) were extremely useful for sex identification (ROC curves, all *P* values ≤ 0.0000099). Especially, the mandibular primary molars (areas under ROC curves [AUCs] between 85.6 and 90.4%, *P* values ≤ 0.0000006) were more useful than the maxillary ones (AUCs between 80.4 and 83.1%, *P* values ≤ 0. 0000099). In the mandible, the first primary molar (maximum AUC = 90.4%) was better than the second molar (maximum AUC = 86.0%). The optimum thresholds for sex determination were reported. Sex dimorphism was significant in buccolingual and mesiodistal crown widths of all the primary molars (all *P* values ≤ 0.000132), but it was seen only in the case of 2 nonmetric traits: Deflecting wrinkle (*P* = 0.001) and Tuberculum intermedium (metaconulid, *P* = 0.029) on the lower Es, taking into account the unilateral and bilateral cases. The occurrence of nonmetric traits was symmetrical between the right and left sides (all *P* values ≥ 0.250). All mesiodistal and two buccolingual molar measurements were as well symmetrical (*P* > 0.1); however, two buccolingual measurements were asymmetrical: in the case of the maxillary E (*P* = 0.0002) and mandibular D (*P* = 0.019). There were three weak-to-moderate correlations between the nonmetric traits of the mandibular second molars (Spearman correlations between 22.7 and 37.5%, *P* values ≤ 0.045). Up to 6 concurrent nonmetric traits were observed in the sample, with 53.6% of the sample showing at least 2 concurrent nonmetric traits at the same time, without any sex dimorphism (*P* = 0.658).

**Conclusion:**

Sex dimorphism exists considerably in primary molars’ sizes, but it is not as prevalent in their nonmetric traits or abnormalities. Primary molars’ crown sizes are useful for sex identification; we calculated optimum cut-off points for this purpose, for the first time.

## Introduction

Human teeth are unique resources for studying genetics, forensics, and anthropology, either in living populations or in non-living ones [1]. Teeth are the tissues that are best protected, because enamel is the hardest tissue in the human body and have the capacity to resist high temperatures and fossilization processes (time, environment, pH, salt, moisture, trace elements) [2]. For this reason, during forensic examinations or archaeological excavations, where not all of a person’s bones have been collected, which is often the case, skulls and teeth are often the only means of identification [3]. As a result, it is possible to differentiate people from different societies using these features; even people who have an unknown identity can be properly assigned to their ethnic community according to the unique morphological characteristics of each society [4].

Sex identification is an important step in reconstructing the biological profile of individuals with unknown identities in forensic medicine [5–8]. The metric sizes and non-metric features of the teeth may provide information about the sex of individuals [9, 10]. There are studies that show differences in metric characteristics of permanent teeth in women and men [5–7, 11]. As a result, in cases where sex recognition is not possible through craniofacial features, metric features of the permanent or primary teeth are used to identify sex [3, 5, 6]. Although studies on sex dimorphism in the primary dentition exist [6, 8], studies on optimum cut-off points for sex identification using the primary dentition are lacking worldwide.

The morphology of deciduous teeth is useful for studying the biological coherence between human populations as well as the biological differentiation between two or more human ethnicities; it also helps to understand human dental development and to recognize the evolutionary differences between deciduous and permanent dentition [4, 12].

Despite their temporary presence in the mouth, the primary teeth provide an excellent model for studying growth diversity [13]. In dental and anthropological texts, deciduous dentition is a unique source of information about evolution [13]. It is also important to study the unique shape and size of deciduous teeth, especially in restorative or endodontic treatments performed by pediatric dentists [14].

Unlike permanent teeth, the metric and non-metric traits and anomalies of primary teeth have not been well documented [14]. Previous studies in genetic analysis have focused exclusively on the relationship between adults using the permanent tooth phenotype. This omits the very important periods of infancy and childhood. This is unfortunate because adult-centered analyses ignore broad topics in the anthropology of children [15, 16].

Besides the importance of this matter as detailed above, there were various reasons that made us conduct this epidemiological study for the first time; they were as follows: there is no study worldwide on the usefulness and cut-off points for sex determination based on the primary dentition; there is no study on metric or non-metric traits and anomalies of deciduous teeth of Iranians; studies worldwide on each of the metric or non-metric traits are only a few and controversial; finally, studies on sex dimorphism or bilateral symmetry or concurrent traits in nonmetric traits are nonexistent or scarce. Its goals were to evaluate the prevalence of various nonmetric traits and anomalies, document buccolingual and mesiodistal crown widths, estimate sex dimorphism in metric and nonmetric traits, and identify potential thresholds for sex identification based on primary molar measurements. The null hypotheses were a lack of any associations between subjects’ sex with any of the metric or nonmetric traits, as well as a lack of cut-off points for sex identification.

## Materials and methods

This cross-sectional epidemiological study was performed on both the left/right sides of the mandibles and maxillae of 110 pediatric patients: 220 maxillary and mandibular dental pretreatment casts of 110 children were selected randomly from private orthodontic and pediatric offices as well as the archives of Ahvaz Dental School.

### Sample and eligibility criteria

The casts had been poured with orthodontic white dental stone prepared by alginate molding. Information about the patient’s sex was extracted from the patient’s file and recorded. The exclusion criteria were the presence of any caries, restoration, stainless steel (SS) crowns, dental wear, missing, any histories of trauma, any congenital or syndromic diseases, any incomplete patient records, as well as a lack of completely erupted primary molars. The eruption of the permanent teeth was not an issue as long as fully erupted, intact primary molars were available.

### Ethics

The study did not collect any identifier or personal information of the patients apart from their anonymously taken sex; the results of the study were completely confidential and will be used only for research purposes. The study was retrospective and used retrospectively taken material; the need for any signed informed consents was waived by the Institutional Review Board of Ahvaz Jundishapur University of Medical Sciences, Ahvaz, Iran (ethics approval code: IR.AJUMS.REC.1399.455). The study protocol and its ethics were approved by the Institutional Review Board of Ahvaz Jundishapur University of Medical Sciences, Ahvaz, Iran (code: IR.AJUMS.REC.1399.455). All methods were performed in accordance with the relevant guidelines and regulations (including the Declaration of Helsinki); all experimental protocols were approved by the Institutional Review Board of Ahvaz Jundishapur University of Medical Sciences, Ahvaz, Iran.

### Sample size

#### Nonmetric

The sample size was determined as 96 patients based on the following formula for non-metric traits:

$$n=\frac{{\textrm{Z}}^2\ast p\ast \left(1-p\right)}{d^2}$$

Where Z was set at 1.96 for a 95% confidence interval, *p* was chosen as 0.5 to obtain the largest sample size within this formula –since this number was not known and among all numbers between zero and 1, this particular number (i.e., 0.5) yields the greatest and the most conservative sample size within this formula; hence it was set at 0.5 in the case of uncertainty; d was the precision and set at 0.1.

#### Metric

This sample size was deemed large enough for the metric measurements as well. The sample size was augmented to 110 patients in order to improve the reliability of the results. Post hoc power calculations for metric measurements showed high powers for all the measurements. For the mesiodistal measurements, all the 8 calculated post hoc powers were ≥ 98.3%, with most of them being 100%. For the buccolingual measurements, all the 8 calculated post hoc powers were ≥ 99.6%, with most of them being 100%.

It should be noted that in this study the evaluations were bilateral in order to improve the reliability and give a more comprehensive picture of the status of each of the traits at the same time. This also allowed to determine the unilaterality and bilaterality of traits. Therefore, each patient provided bilateral maxillary and mandibular first and second molars.

### Assessments

All the assessments were performed by a trained and calibrated last-year dental student, who used a magnifying glass to ensure appropriate accuracy. Any questionable cases would be also checked by an experienced orthodontist.

#### Nonmetric traits, variations, or anomalies

All left and right primary molars were carefully examined for the presence or absence of several nonmetric traits or anomalies on the primary first and second molars of the maxilla and the mandible. Crown characteristics were observed based on the ASUDAS (Arizona State University Dental Anthropology System) method. The goal in this method is to obtain reproducible results beyond the mere presence or absence of an anatomical feature. This means that based on the intensity of prominence and visibility of the features, a number is recorded for each anatomical feature [17]. The traits/anomalies were the accessory cusp on the upper D, the accessory cusp on the lower D, the fifth cusp on the upper E, the Carabelli’s cusp, the protostylid, the fifth cusp on the lower E, the sixth cusp on the lower E, the tuberculum intermedium (metaconulid) on the lower E, and deflecting wrinkles. The definition of the evaluated traits is as follows:

1. Accessory cusp on both the mandibular or maxillary D: A supernumerary cusp on the mesial or distal marginal ridges of the maxillary and mandibular first molars.
2. The fifth cusp on the maxillary E: An extra cusp between the distobuccal and distopalatal cusps on the primary maxillary second molars.
3. The Carabelli’s cusp: A small cusp on the mesiopalatal surface of the primary maxillary second molar.
4. Protostylid: An extra cusp on the mesial half of the buccal surface of the mandibular primary second molar.
5. The fifth cusp on the mandibular E: A small supernumerary cusp between the distobuccal cusp and the distolingual cusp of the lower deciduous second molar.
6. The sixth cusp on the mandibular E: An additional cusp on the distal marginal ridge between the distobuccal and distolingual cusps of the primary lower second molar.
7. The tuberculum intermedium (metaconulid or the seventh cusp) on the mandibular E: An extra cusp between the mesiolingual and distolingual cusps of the lower primary second molar.
8. The deflecting wrinkle: An additional middle ridge on the mesiolingual cusp of the primary lower second molar.

#### Metric measurements

The mesiodistal and buccolingual widths of the primary molars were measured by a digital caliper (Insize, China) with an accuracy of 0.01 mm. The mesiodistal width of the crown was defined as the longest distance from the mesial to the distal contact points, parallel to the occlusal surface of the tooth; i.e., the longest distance between the contact points while the caliper is parallel to the occlusal surface and the buccal surface. If there were no adjacent teeth, the contact point would be determined from the anatomy of the tooth. The buccolingual width of the tooth crown was defined as the longest distance between the buccal (or labial) and lingual/palatal contact points, perpendicular to the mesiodistal dimension [5–7, 10].

### Intraobserver error

After 1 month, 36 cases (144 quadrants) were randomly reevaluated. All the metric and non-metric traits were reassessed by the same observer. All the metric and non-metric properties of the teeth were re-evaluated. The intraobserver agreement was perfect for the mesiodistal dimensions of all the first and second molars on the right or left sides of the maxilla or the mandible (all 8 intraclass correlation coefficients or ICCs were either 99.9% or 100%, all 8 *P* values = 0.000000). The intraobserver agreement was excellent for the buccolingual dimensions of 7 tooth types (all except the maxillary right second molar). The 7 calculated ICC values for these teeth were either 99.9% or 100%, with all the 7 *P* values = 0.000000. For the maxillary right second molar, the ICC was 78.5% (*P* = 0.0002).

For the non-metric traits, the intrarater agreements –indicated by Kappa values– were as follows: The accessory cusp on the upper D (Kappa = 85.5%), the accessory cusp on lower D (100%), the fifth cusp on the upper E (93.2%), the Carabelli’s cusp (92.5%), Protostylid (93.7%), the fifth cusp on the lower E (100%), the sixth cusp on the lower E (91.3%), the seventh cusp on the lower E (81.1%), and deflecting wrinkle (94.1%); the *P* values for all these 9 Kappa values were < 0.0005.

### Statistical analyses

Descriptive statistics and 95% confidence intervals (CIs) were computed for the variables. The statistics of patients with different numbers of concurrent traits were evaluated [18]. The normality of the metric measurements was confirmed through the evaluation of histograms and q-q plots as well as noting the central limit theorem. Sex dimorphism was evaluated for nonmetric dental traits using a Fisher exact test and a chi-square test. An independent-samples t-test was used to evaluate the existence of sex dimorphism in the buccolingual and mesiodistal widths of the teeth. A receiver-operator characteristic (ROC) curve was used to assess the usefulness of the sizes of the teeth for forensic sex determination (based on areas under the curve (AUC)) as well as the cut-off points for differentiating boys from girls. For this purpose, first, the left and right sides were combined by either taking the average of left and right sides (when both values were present) or by using left or right values (when the contralateral side was not available). A paired t-test was used to compare the metric measurements of the teeth on the right and left sides; a Pearson correlation coefficient was used to evaluate the correlation between the sizes on the right and left sides. A McNemar test was used to examine right/left symmetry for nonmetric traits. A Spearman correlation coefficient was used to examine correlations between different nonmetric traits in the same teeth. The software in use was SPSS 27 (IBM, Armonk, NY, USA). The level of significance was set at 0.05.

## Results

There were 59 girls and 51 boys. The children’s age ranged between 6 and 12 years (average: 9 years).

### Metric traits

Overall, the mean (SD) mesiodistal width of the primary maxillary first molar was 7.22 ± 0.60 mm. The buccolingual size of the same tooth was 8.90 ± 0.74 mm. For the upper E, the mean mesiodistal and buccolingual sizes were 8.98 ± 0.71 mm and 9.97 ± 0.61 mm, respectively. For the mandibular D, the mean mesiodistal and buccolingual sizes were 8.07 ± 0.58 mm and 7.83 ± 0.62 mm, respectively. For the lower E, the mean mesiodistal and buccolingual sizes were 10.03 ± 0.69 mm and 9.40 ± 0.78 mm, respectively. The coefficients of variation showed a subtle dispersity for all measurements (Tables 1, 2 and 3).

**Table 1:** Descriptive statistics and 95% CIs for the mesiodistal and buccolingual dimensions of the teeth (mm). The *P* values are calculated using the t-test

Jaw	Side	Tooth	Width	Sex	N	Mean	SD	CV (%)	95% CI		Min	Max	*P*
**Maxilla**	**Right**	**1st molar**	**Mesiodistal**	**Female**	31	6.91	0.49	7.1	6.73	7.09	6.15	8.16	0.000010
				**Male**	30	7.52	0.49	6.5	7.34	7.70	6.10	8.36	
				**Both**	61	7.21	0.57	7.9	7.06	7.36	6.10	8.36	
			**Buccolingual**	**Female**	31	8.57	0.54	6.3	8.37	8.76	7.23	9.48	0.000015
				**Male**	30	9.30	0.66	7.1	9.05	9.54	7.44	10.01	
				**Both**	61	8.92	0.70	7.8	8.74	9.10	7.23	10.01	
		**2nd molar**	**Mesiodistal**	**Female**	50	8.65	0.50	5.8	8.51	8.79	7.75	9.96	0.000000
				**Male**	41	9.36	0.68	7.3	9.15	9.57	7.96	10.53	
				**Both**	91	8.97	0.68	7.6	8.83	9.11	7.75	10.53	
			**Buccolingual**	**Female**	50	9.67	0.46	4.8	9.53	9.80	8.89	10.99	0.000000
				**Male**	41	10.27	0.58	5.6	10.09	10.46	8.78	11.16	
				**Both**	91	9.94	0.60	6.0	9.81	10.06	8.78	11.16	
	**Left**	**1st molar**	**Mesiodistal**	**Female**	26	6.92	0.53	7.7	6.70	7.13	6.19	8.31	0.000132
				**Male**	24	7.58	0.59	7.8	7.33	7.82	5.60	8.56	
				**Both**	50	7.23	0.65	9.0	7.05	7.42	5.60	8.56	
			**Buccolingual**	**Female**	26	8.44	0.55	6.5	8.22	8.67	7.20	9.66	0.000017
				**Male**	24	9.34	0.77	8.2	9.02	9.67	7.34	10.22	
				**Both**	50	8.88	0.80	9.0	8.65	9.10	7.20	10.22	
		**2nd molar**	**Mesiodistal**	**Female**	41	8.60	0.54	6.3	8.43	8.77	7.68	9.98	0.000000
				**Male**	36	9.46	0.69	7.3	9.22	9.69	7.75	10.52	
				**Both**	77	9.00	0.75	8.3	8.83	9.17	7.68	10.52	
			**Buccolingual**	**Female**	41	9.72	0.50	5.1	9.56	9.87	8.86	10.83	0.000003
				**Male**	36	10.34	0.59	5.7	10.14	10.54	8.75	11.08	
				**Both**	77	10.01	0.62	6.2	9.87	10.15	8.75	11.08	
**Mandible**	**Right**	**1st molar**	**Mesiodistal**	**Female**	24	7.67	0.50	6.5	7.46	7.88	6.70	9.04	0.000000
				**Male**	25	8.48	0.38	4.5	8.32	8.63	7.68	8.98	
				**Both**	49	8.08	0.60	7.4	7.91	8.25	6.70	9.04	
			**Buccolingual**	**Female**	24	7.50	0.47	6.3	7.31	7.70	6.44	8.42	0.000002
				**Male**	25	8.27	0.53	6.4	8.06	8.49	7.45	9.23	
				**Both**	49	7.90	0.63	8.0	7.72	8.08	6.44	9.23	
		**2nd molar**	**Mesiodistal**	**Female**	31	9.54	0.44	4.6	9.38	9.70	8.78	10.81	0.000000
				**Male**	33	10.46	0.63	6.0	10.24	10.69	9.07	11.34	
				**Both**	64	10.02	0.71	7.1	9.84	10.19	8.78	11.34	
			**Buccolingual**	**Female**	31	8.88	0.40	4.5	8.73	9.02	8.18	9.78	0.000000
				**Male**	33	9.93	0.75	7.6	9.66	10.19	7.99	11.00	
				**Both**	64	9.42	0.80	8.5	9.22	9.62	7.99	11.00	
	**Left**	**1st molar**	**Mesiodistal**	**Female**	22	7.66	0.48	6.3	7.45	7.87	6.68	8.77	0.000000
				**Male**	26	8.41	0.39	4.6	8.25	8.57	7.71	8.95	
				**Both**	48	8.07	0.57	7.1	7.90	8.23	6.68	8.95	
			**Buccolingual**	**Female**	22	7.37	0.43	5.8	7.18	7.56	6.56	8.22	0.000008
				**Male**	26	8.10	0.55	6.8	7.88	8.32	7.17	9.33	
				**Both**	48	7.77	0.62	8.0	7.59	7.94	6.56	9.33	
		**2nd molar**	**Mesiodistal**	**Female**	34	9.66	0.47	4.9	9.49	9.82	8.80	10.80	0.000000
				**Male**	36	10.41	0.62	6.0	10.20	10.62	8.92	11.56	
				**Both**	70	10.04	0.67	6.7	9.88	10.20	8.80	11.56	
			**Buccolingual**	**Female**	34	8.92	0.44	4.9	8.76	9.07	8.15	9.96	0.000000
				**Male**	36	9.83	0.75	7.6	9.58	10.09	8.26	11.01	
				**Both**	70	9.39	0.77	8.2	9.20	9.57	8.15	11.01	

*CV* coefficient of variation, *SD* standard deviation, *CI* confidence interval, *Min* minimum, *Max* maximum

**Table 2:** Descriptive statistics and 95% CIs for the mesiodistal and buccolingual dimensions of the teeth on the right and left sides (mm) regardless of sex

Jaw	Tooth	Width	Side	N	Mean	SD	CV (%)	95% CI		Min	Max
**Maxilla**	**D**	**Mesiodistal**	**Right**	61	7.21	0.57	7.9	7.06	7.36	6.10	8.36
			**Left**	50	7.23	0.65	9.0	7.05	7.42	5.60	8.56
			**Total**	111	7.22	0.60	8.3	7.11	7.33	5.60	8.56
		**Buccolingual**	**Right**	61	8.92	0.70	7.8	8.74	9.10	7.23	10.01
			**Left**	50	8.88	0.80	9.0	8.65	9.10	7.20	10.22
			**Total**	111	8.90	0.74	8.3	8.76	9.04	7.20	10.22
	**E**	**Mesiodistal**	**Right**	91	8.97	0.68	7.6	8.83	9.11	7.75	10.53
			**Left**	77	9.00	0.75	8.3	8.83	9.17	7.68	10.52
			**Total**	168	8.98	0.71	7.9	8.88	9.09	7.68	10.53
		**Buccolingual**	**Right**	91	9.94	0.60	6.0	9.81	10.06	8.78	11.16
			**Left**	77	10.01	0.62	6.2	9.87	10.15	8.75	11.08
			**Total**	168	9.97	0.61	6.1	9.88	10.06	8.75	11.16
**Mandible**	**D**	**Mesiodistal**	**Right**	49	8.08	0.60	7.4	7.91	8.25	6.70	9.04
			**Left**	48	8.07	0.57	7.1	7.90	8.23	6.68	8.95
			**Total**	97	8.07	0.58	7.2	7.96	8.19	6.68	9.04
		**Buccolingual**	**Right**	49	7.90	0.63	8.0	7.72	8.08	6.44	9.23
			**Left**	48	7.77	0.62	8.0	7.59	7.94	6.56	9.33
			**Total**	97	7.83	0.62	7.9	7.71	7.96	6.44	9.33
	**E**	**Mesiodistal**	**Right**	64	10.02	0.71	7.1	9.84	10.19	8.78	11.34
			**Left**	70	10.04	0.67	6.7	9.88	10.20	8.80	11.56
			**Total**	134	10.03	0.69	6.9	9.91	10.15	8.78	11.56
		**Buccolingual**	**Right**	64	9.42	0.80	8.5	9.22	9.62	7.99	11.00
			**Left**	70	9.39	0.77	8.2	9.20	9.57	8.15	11.01
			**Total**	134	9.40	0.78	8.3	9.27	9.54	7.99	11.01

*CV* coefficient of variation, *SD* standard deviation, *CI* confidence interval, *Min* minimum, *Max* maximum

**Table 3:** Descriptive statistics and 95% CIs for the mesiodistal and buccolingual dimensions of the teeth (mm) after merging the right and left sides. The *P* values are calculated using the t-test

Jaw	Tooth	Width	Sex	N	Mean	SD	CV (%)	95% CI		Min	Max	*P*
**Maxilla**	**1st molar**	**Mesiodistal**	**Female**	37	6.926	0.468	6.8	6.770	7.082	6.150	8.235	0.000009
			**Male**	34	7.501	0.540	7.2	7.313	7.689	6.100	8.460	
			**Total**	71	7.201	0.577	8.0	7.065	7.338	6.100	8.460	
		**Buccolingual**	**Female**	37	8.530	0.509	6.0	8.361	8.700	7.215	9.570	0.000001
			**Male**	34	9.321	0.728	7.8	9.068	9.575	7.340	10.110	
			**Total**	71	8.909	0.736	8.3	8.735	9.083	7.215	10.110	
	**2nd molar**	**Mesiodistal**	**Female**	54	8.615	0.496	5.8	8.480	8.750	7.680	9.935	0.000000
			**Male**	43	9.418	0.681	7.2	9.208	9.628	7.865	10.530	
			**Total**	97	8.971	0.707	7.9	8.828	9.113	7.680	10.530	
		**Buccolingual**	**Female**	54	9.696	0.461	4.8	9.570	9.822	8.885	10.910	0.000000
			**Male**	43	10.331	0.573	5.5	10.155	10.508	8.925	11.110	
			**Total**	97	9.978	0.601	6.0	9.856	10.099	8.885	11.110	
**Mandible**	**1st molar**	**Mesiodistal**	**Female**	29	7.636	0.463	6.1	7.460	7.812	6.690	8.905	0.000000
			**Male**	31	8.426	0.401	4.8	8.279	8.573	7.680	8.960	
			**Total**	60	8.044	0.585	7.3	7.893	8.195	6.690	8.960	
		**Buccolingual**	**Female**	29	7.432	0.458	6.2	7.257	7.606	6.530	8.420	0.000000
			**Male**	31	8.209	0.543	6.6	8.010	8.408	7.375	9.280	
			**Total**	60	7.833	0.635	8.1	7.669	7.997	6.530	9.280	
	**2nd molar**	**Mesiodistal**	**Female**	40	9.621	0.454	4.7	9.475	9.766	8.790	10.805	0.000000
			**Male**	39	10.421	0.603	5.8	10.225	10.616	9.035	11.425	
			**Total**	79	10.016	0.665	6.6	9.867	10.165	8.790	11.425	
		**Buccolingual**	**Female**	40	8.891	0.414	4.7	8.759	9.024	8.165	9.870	0.000000
			**Male**	39	9.845	0.755	7.7	9.600	10.090	8.155	10.990	
			**Total**	79	9.362	0.771	8.2	9.189	9.535	8.155	10.990	

*CV* coefficient of variation, *SD* standard deviation, *CI* confidence interval, *Min* minimum, *Max* maximum

#### Sex dimorphism

The independent-sample t-test showed significant sex dimorphism in all the mesiodistal and buccolingual sizes of all the primary molars either in each side separately (Table 1).

#### Bilateral symmetry

According to the paired t-test, no significant difference was observed between the mesiodistal measurements of the teeth on the right versus left sides (Table 2; *P* = 0.277 for the maxillary D; *P* = 0.168 for the maxillary E; *P* = 0.144 for the mandibular D; *P* = 0.774 for the mandibular E). Nevertheless, the buccolingual measurements were slightly yet statistically significantly different in the case of the primary maxillary second molar and the primary mandibular first molar (Table 2; *P* = 0.821 for the maxillary D; *P* = 0.0002 for the maxillary E; *P* = 0.019 for the mandibular D; *P* = 0.283 for the mandibular E).

#### Correlations

The Pearson coefficient showed excellent correlations between the right and left sides in terms of the mesiodistal dimensions of the maxillary and mandibular first and second molars (all the 4 correlation coefficients were between 91.1 and 97.3%, all 4 *P* values < 0.0000000001). Similarly, there existed excellent correlations between the right and left sides in terms of the buccolingual dimensions of the maxillary and mandibular first and second molars (all the 4 correlation coefficients were between 92.2 and 97.8%, all 4 *P* values < 0.0000000001).

#### ROC curves

**Fig. 1:**
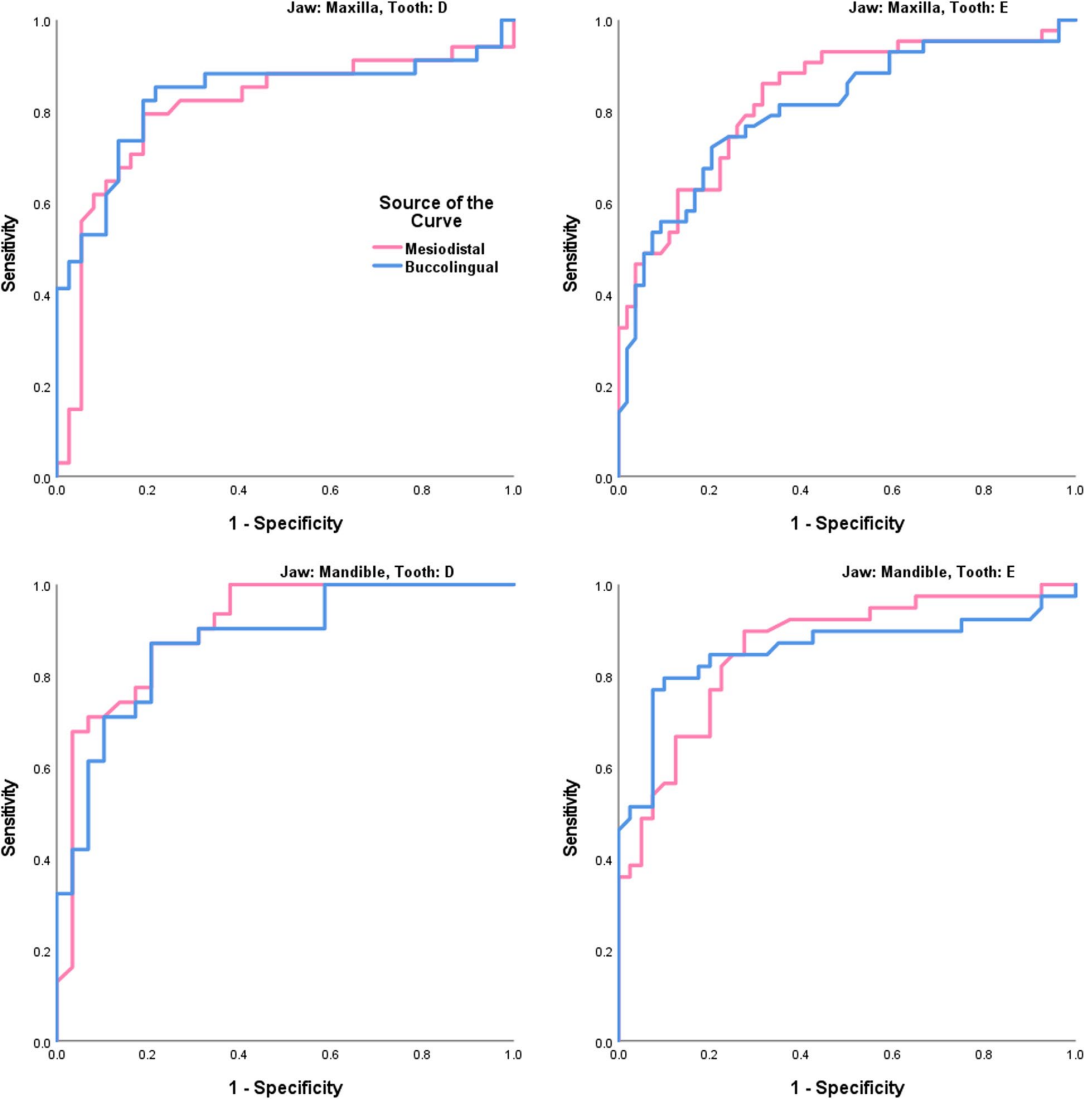
The ROC curves showing very large areas under the curve for sex determination. Curves for the mesiodistal dimension are in pink while curves pertaining to the buccolingual size are in blue

After combining both sides by taking their averages, the independent-sample t-test showed significant sex dimorphism in all the mesiodistal and buccolingual sizes of all the primary molars (Table 3). The areas under the ROC curves indicated a very high usefulness of all the 4 teeth (both molars of both jaws) in differentiating boys and girls (Table 4, Fig. 1).

**Table 4:** The areas under the ROC curves as well as the optimum cut-off points for determining the sex based on dental measurements

Jaw	Tooth	Dimensions	AUC	SE	*P*	95% CI for AUC		Cut-off (mm)
**Maxilla**	**D**	**Mesiodistal**	0.805	0.057	0.0000099	0.693	0.917	7.4925
		**Buccolingual**	0.830	0.054	0.0000017	0.725	0.936	8.9075
	**E**	**Mesiodistal**	0.831	0.043	0.0000000	0.747	0.914	8.7550
		**Buccolingual**	0.804	0.046	0.0000003	0.714	0.894	10.3100
**Mandible**	**D**	**Mesiodistal**	0.904	0.040	0.0000001	0.827	0.982	7.9850
		**Buccolingual**	0.874	0.045	0.0000006	0.787	0.962	7.7200
	**E**	**Mesiodistal**	0.860	0.042	0.0000000	0.777	0.942	9.8350
		**Buccolingual**	0.856	0.047	0.0000001	0.764	0.949	9.3950

*AUC* area under the curve, *SE* standard error, *CI* confidence interval

#### Cut-off points for sex identification

The optimum thresholds for sex determination are reported in Table 4. The mandibular teeth showed a stronger sensitivity and specificity for sex identification compared to the maxillary teeth. In the mandible, the first primary molar was more useful than the second molar.

### Nonmetric traits or variations

#### Prevalences

The prevalences of the examined nonmetric dental traits or anomalies in patients were as follows: the Accessory cusp on the upper D (5.6%), the Accessory cusp on the lower D (18.3%), the Fifth cusp on the upper E (12.4%), the Carabelli’s cusp on the maxillary E (33%), the Protostylid on the mandibular E (26.6%), the Fifth cusp on the lower E (100%), the Sixth cusp on the lower E (17.7%), the Tuberculum intermedium (metaconulid) on the lower E (27.8%), and the Deflecting wrinkle on the mandibular E (35.4%).

### Sex dimorphism considering the sides

The Fisher test showed a significant sex dimorphism only in the case of deflecting wrinkle on mandibular second molars of both sides, being significantly more prevalent in men (Table 5).

**Table 5:** Prevalences of different traits and anomalies in each side of each jaw, in males and females. The *P* values are calculated using the Fisher exact test

Jaw	Side	Tooth	Trait		Sex (%)		Total prevalence (%)	*P*
					Female	Male		
**Maxilla**	**Right**	**D**	**Accessory cusp on the upper D**	**Absent**	29 (47.5)	28 (45.9)	57 (93.4)	1.0
				**Present**	2 (3.3)	2 (3.3)	4 (6.6)	
	**Left**	**D**	**Accessory cusp on the upper D**	**Absent**	24 (48)	23 (46)	47 (94)	1.0
				**Present**	2 (4)	1 (2)	3 (6)	
**Maxilla**	**Right**	**E**	**Carabelli’s cusp on the upper E**	**Absent**	34 (37.4)	27 (29.7)	61 (67)	1.0
				**Present**	16 (17.6)	14 (15.4)	30 (33)	
	**Left**	**E**	**Carabelli’s cusp on the upper E**	**Absent**	28 (36.4)	28 (36.4)	56 (72.7)	0.444
				**Present**	13 (16.9)	8 (10.4)	21 (27.3)	
**Mandible**	**Right**	**E**	**Protostylid on the lower E**	**Absent**	24 (37.5)	22 (34.4)	46 (71.9)	0.410
				**Present**	7 (10.9)	11 (17.2)	18 (28.1)	
	**Left**	**E**	**Protostylid on the lower E**	**Absent**	27 (38.6)	25 (35.7)	52 (74.3)	0.417
				**Present**	7 (10)	11 (15.7)	18 (25.7)	
**Mandible**	**Right**	**E**	**Deflecting wrinkle on the lower E**	**Absent**	24 (37.5)	14 (21.9)	38 (59.4)	**0.006**
				**Present**	7 (10.9)	19 (29.7)	26 (40.6)	
	**Left**	**E**	**Deflecting wrinkle on the lower E**	**Absent**	30 (42.9)	16 (22.9)	46 (65.7)	**0.0001**
				**Present**	4 (5.7)	20 (28.6)	24 (34.3)	
**Maxilla**	**Right**	**E**	**Fifth cusp on the upper E**	**Absent**	45 (49.5)	34 (37.4)	79 (86.8)	0.364
				**Present**	5 (5.5)	7 (7.7)	12 (13.2)	
	**Left**	**E**	**Fifth cusp on the upper E**	**Absent**	38 (49.4)	33 (42.9)	71 (92.2)	1.0
				**Present**	3 (3.9)	3 (3.9)	6 (7.8)	
**Mandible**	**Right**	**D**	**Accessory cusp on the lower D**	**Absent**	19 (38.8)	21 (42.9)	40 (81.6)	0.725
				**Present**	5 (10.2)	4 (8.2)	9 (18.4)	
	**Left**	**D**	**Accessory cusp on the lower D**	**Absent**	16 (33.3)	23 (47.9)	39 (81.3)	0.267
				**Present**	6 (12.5)	3 (6.3)	9 (18.8)	
**Mandible**	**Right**	**E**	**Fifth cusp on the lower E**	**Present**	31 (48.4)	33 (51.6)	64 (100)	NA
	**Left**	**E**	**Fifth cusp on the lower E**	**Absent**	1 (1.4)	0 (0)	1 (1.4)	0.486
				**Present**	33 (47.1)	36 (51.4)	69 (98.6)	
**Mandible**	**Right**	**E**	**Sixth cusp on the lower E**	**Absent**	24 (37.5)	28 (43.8)	52 (81.3)	0.531
				**Present**	7 (10.9)	5 (7.8)	12 (18.8)	
	**Left**	**E**	**Sixth cusp on the lower E**	**Absent**	28 (40)	30 (42.9)	58 (82.9)	1.0
				**Present**	6 (8.6)	6 (8.6)	12 (17.1)	
**Mandible**	**Right**	**E**	**Tuberculum intermedium (metaconulid) on the lower E**	**Absent**	17 (26.6)	26 (40.6)	43 (67.2)	0.062
				**Present**	14 (21.9)	7 (10.9)	21 (32.8)	
	**Left**	**E**	**Tuberculum intermedium (metaconulid) on the lower E**	**Absent**	24 (34.3)	29 (41.4)	53 (75.7)	0.408
				**Present**	10 (14.3)	7 (10)	17 (24.3)	

*NA* not applicable, i.e., a statistical comparison was not applicable, because both sexes similarly showed a full presence of the fifth cusp on mandibular right lower Es. Significant *P* values in bold font

#### Bilateral symmetry

The McNemar test showed right-left symmetries for the presence of all the assessed nonmetric traits (Table 6).

**Table 6:** The evaluation of the symmetry between the prevalences of the traits on the right versus left sides. The *P* values are calculated using the McNemar test

Trait	Jaw	Tooth	Right Side		Left side		Total	*P*
					Absent	Present		
**Accessory cusp on upper D**	**Maxilla**	**D**	**Right**	**Absent**	37	0	37	1.0
				**Present**	0	3	3	
			**Total**		37	3	40	
**Accessory cusp on lower D**	**Mandible**	**D**	**Right**	**Absent**	27	2	29	1.0
				**Present**	1	7	8	
			**Total**		28	9	37	
**Fifth cusp on upper E**	**Maxilla**	**E**	**Right**	**Absent**	62	0	62	0.250
				**Present**	3	6	9	
			**Total**		65	6	71	
**Carabelli’s cusp**	**Maxilla**	**E**	**Right**	**Absent**	50	0	50	0.500
				**Present**	2	19	21	
			**Total**		52	19	71	
**Protostylid**	**Mandible**	**E**	**Right**	**Absent**	37	1	38	1.0
				**Present**	2	15	17	
			**Total**		39	16	55	
**Fifth cusp on lower E**	**Mandible**	**E**	**Right**	**Present**	1	54	55	NA
			**Total**		1	54	55	
**Sixth cusp on lower E**	**Mandible**	**E**	**Right**	**Absent**	44	1	45	1.0
				**Present**	0	10	10	
			**Total**		44	11	55	
**Tuberculum intermedium (metaconulid) on lower E**	**Mandible**	**E**	**Right**	**Absent**	36	0	36	0.250
				**Present**	3	16	19	
			**Total**		39	16	55	
**Deflecting wrinkle**	**Mandible**	**E**	**Right**	**Absent**	30	0	30	0.250
				**Present**	3	22	25	
			**Total**		33	22	55	

*NA* not applicable because of the lack of this trait on one of the sides

#### Prevalences of bilateral and unilateral cases

Table 7 summarizes the bilateral and unilateral cases in males, females, and the total sample after combining the right and left sides and calculating the unilateral and bilateral prevalences in each patient.

**Table 7:** Prevalences of different traits and anomalies in both sides of jaws in boys and girls. The *P* values are calculated using the chi-square test

Jaw	Tooth	Trait		Sex (%)		Total prevalence (%)	*P*
				Female	Male		
**Maxilla**	**D**	**Accessory cusp on the upper D**	**Absent**	35 (49.3)	32 (45.1)	67 (94.4)	0.511
			**Unilateral**	0 (0)	1 (1.4)	1 (1.4)	
			**Bilateral**	2 (2.8)	1 (1.4)	3 (4.2)	
**Maxilla**	**E**	**Carabelli’s cusp on the upper E**	**Absent**	36 (37.1)	29 (29.9)	65 (67)	0.972
			**Unilateral**	7 (7.2)	6 (6.2)	13 (13.4)	
			**Bilateral**	11 (11.3)	8 (8.2)	19 (19.6)	
**Mandible**	**E**	**Protostylid on the lower E**	**Absent**	31 (39.2)	27 (34.2)	58 (73.4)	0.273
			**Unilateral**	4 (5.1)	2 (2.5)	6 (7.6)	
			**Bilateral**	5 (6.3)	10 (12.7)	15 (19)	
**Mandible**	**E**	**Deflecting wrinkle on the lower E**	**Absent**	33 (41.8)	18 (22.8)	51 (64.6)	**0.001**
			**Unilateral**	3 (3.8)	3 (3.8)	6 (7.6)	
			**Bilateral**	4 (5.1)	18 (22.8)	22 (27.8)	
**Maxilla**	**E**	**Fifth cusp on the upper E**	**Absent**	49 (50.5)	36 (37.1)	85 (87.6)	0.490
			**Unilateral**	2 (2.1)	4 (4.1)	6 (6.2)	
			**Bilateral**	3 (3.1)	3 (3.1)	6 (6.2)	
**Mandible**	**D**	**Accessory cusp on the lower D**	**Absent**	22 (36.7)	27 (45)	49 (81.7)	0.452
			**Unilateral**	3 (5)	1 (1.7)	4 (6.7)	
			**Bilateral**	4 (6.7)	3 (5)	7 (11.7)	
**Mandible**	**E**	**Fifth cusp on the lower E**	**Unilateral**	16 (20.3)	9 (11.4)	25 (31.6)	0.106
			**Bilateral**	24 (30.4)	30 (38)	54 (68.4)	
**Mandible**	**E**	**Sixth cusp on the lower E**	**Absent**	32 (40.5)	33 (41.8)	65 (82.3)	0.606
			**Unilateral**	3 (3.8)	1 (1.3)	4 (5.1)	
			**Bilateral**	5 (6.3)	5 (6.3)	10 (12.7)	
**Mandible**	**E**	**Tuberculum intermedium (metaconulid) on the lower E**	**Absent**	25 (31.6)	32 (40.5)	57 (72.2)	**0.029**
			**Unilateral**	6 (7.6)	0 (0)	6 (7.6)	
			**Bilateral**	9 (11.4)	7 (8.9)	16 (20.3)	

Significant *P* values in bold font

#### Sex dimorphism in patients

**Fig. 2:**
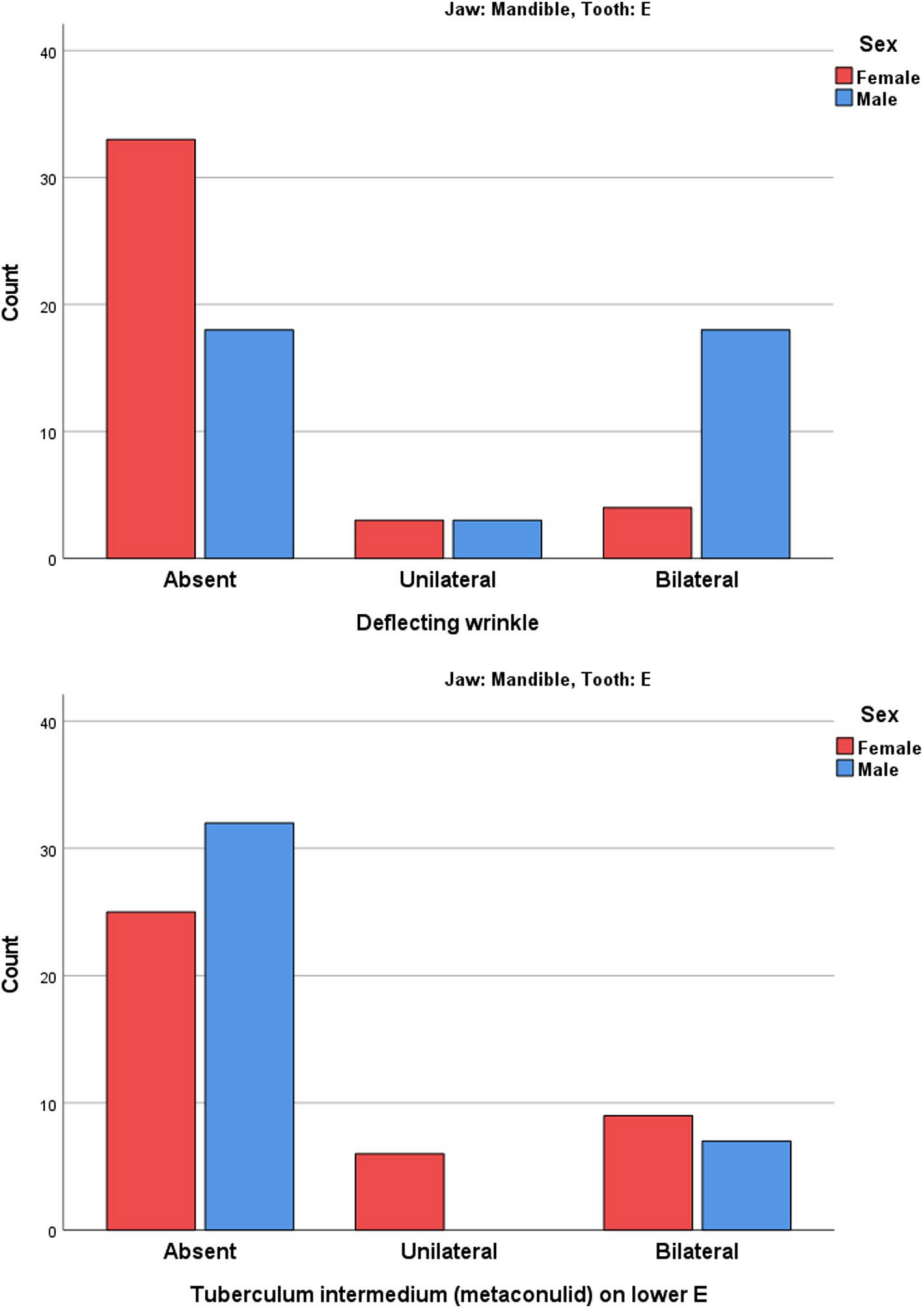
The prevalence of deflecting wrinkles and metaconulids in mandibular second molars

After calculating the unilateral and bilateral cases in each patient, it was observed that deflecting wrinkle was more common in boys while tuberculum intermedium (metaconulid) on the lower E was more common in girls (Table 7, Fig. 2).

#### Correlations across traits

The Spearman correlation showed a few weak-to-moderate correlations between traits and their severity (unilateral versus bilateral) on lower Es (Table 8). The upper Es did not show a significant correlation between the Carabelli and fifth cusps (*P* = 0.517).

**Table 8:** The results of the Spearman correlation coefficient, testing correlations among the occurrences of the traits (coded as absent = 0, unilateral = 1, or bilateral = 2) in the primary mandibular second molars. N for each correlation is 79

		Protostylid	Fifth cusp	Sixth cusp	Metaconulid
**Fifth cusp on lower E**	**Rho**	0.208			
	***P***	0.066			
**Sixth cusp on lower E**	**Rho**	0.045	0.129		
	***P***	0.693	0.259		
**Tuberculum intermedium (metaconulid) on lower E**	**Rho**	0.15	**0.227**	**0.247**	
	***P***	0.188	**0.045**	**0.028**	
**Deflecting wrinkle on lower E**	**Rho**	0.174	**0.375**	0.188	0.195
	***P***	0.124	**0.0007**	0.098	0.085

Significant correlations in bold font. Rho, Spearman coefficient

#### Concurrent occurrences

There were no cases with 7 or more concurrent traits; the maximum number was 6 concurrent traits. Of the 110 patients, 21 patients or 19.1% had 0 traits, 30 patients or 27.3% of the sample had only 1 trait, 18 cases or 16.4% had 2 concurrent traits simultaneously, 21 patients or 19.1% had 3 concurrent traits, 9 patients or 8.2% had 4 concurrent traits, 8 patients or 7.3% had 5 concurrent traits, and 3 cases or 2.7% of the sample had concurrent 6 traits. Hence, 59 cases or 53.6% of the sample had at least 2 concurrent traits or anomalies. Of these 59 cases, 30 were girls and 29 were boys; there was no significant sex dimorphism in terms of the concurrent occurrences of nonmetric traits, considering 17 girls and 13 boys with only 1 trait (Fisher exact test, *P* = 0.658).

## Discussion

In this study, there were weak correlations among certain nonmetric traits of the mandibular Es; also, concurrent nonmetric traits were rather prevalent: more than half of the sample showed at least two traits per patient. These concurrent traits did not show sex dimorphism. Non-metric traits were symmetrical –with similar distributions on the left and right sides; most metric sizes were symmetrical as well, except for buccolingual measurements of the maxillary E and mandibular D which were slightly, still statistically significantly, asymmetrical. Sex dimorphism was found to be very vivid in metric sizes but not much prevalent in nonmetric traits (only two traits showed sex dimorphism). The findings of this study suggested the usefulness of the primary molars’ mesiodistal and buccolingual widths for sex identification. This was stronger in the mandibular primary molars, especially the first molar. The optimum cutoff points were calculated for sex determination in this particular ethnic group: teeth with sizes above the calculated cutoffs more likely belong to boys, while teeth with sizes below this cutoff are more likely indicating a girl.

The shape, size and number of teeth in human dentition are changing at different rates between various geographical and ethnic groups [19]. In order to better understand the mechanism and importance of these changes, they should be examined from three perspectives: genetics, evolution and morphology [19]. Hence, human teeth provide useful information for studying human populations and a basis for comparing their genetic origin. Due to the morphological characteristics of teeth and their frequency, gender differences and bilateral symmetry, human ethnicities can be classified into different evolutionary classes. This is possible because teeth are usually preserved even in severe conditions [13, 20–22]. Teeth can also be used to assess the biological relationship between populations [23]. Morphological features of the teeth provide information about ancestral relationships between different species [1]. Like other biological features, the morphological features of teeth also show inheritance in human populations [24]. Variation in characteristics of dentition have been observed among different populations [1, 25]. Different environmental, cultural and racial factors affect the morphology and size of teeth [1]. The morphology of the teeth depends to some extent on the genetics of individuals, and it is thought that both deciduous and permanent dental systems are equally dependent on genetic factors [20, 21, 26, 27]. To the best of our knowledge, no study worldwide has estimated optimal cut-off points of primary teeth for identifying the sex of a child. However, other aspects of sex dimorphism and sex identification using the primary dentition have been examined –although not in Iran. The morphology of teeth may vary among different populations [25]. This also includes the different degrees of the appearance of teeth between the sexes (sexual dimorphism) in the primary dentition which has been observed across different populations [6, 8, 28–33]. The study by Shankar et al. [32] showed that the tooth dimensions of boys were larger than girls. Shankar et al. in another study [31] observed a significant difference between the sexes in the mean mesiodistal dimensions of the right canine and the right and left first molars and the buccolingual dimensions of first molars in the right side of the maxilla. Similar reports were published by almost any other group who evaluated this matter in terms of buccolingual and/or mesiodistal dimensions [6, 8, 28–34]. The largest reported sex difference in the appearance of deciduous teeth might be in the European Caucasian population of Burlington in the United States with a difference of 4% [30], followed by the African-American population with 3.0% [35], the Australian natives with 2.5% [36], and the Taiwanese-Chinese population with 1.1% [37, 38]. Previous studies have also shown that the dimensions of teeth in boys are larger than girls [33]. This finding was consistent with the present study because all dental dimensions in boys were significantly larger than girls. Differences between the sexes, taking into account differences between populations, can be used in forensic dentistry and archeology to identify the biological sex of skeletal remains of children. Of course, this is one of the elements of the child’s skeleton that show such marked dimorphism –other elements including the hip bone [39] and the mandible [40].

In the permanent dentition, sexual dimorphism might not be prevalent in non-metric traits or anomalies [18]. In the present study, there was a significant difference between the two sexes in the prevalence of deflecting wrinkle and metaconulid. In this regard, Adler and Donlon [28] found no significant sex dimorphism. The differences in the results may of course be attributed to genetic, ethnic, and environmental diversity in the populations studied in various studies. The nonmetric morphology of the primary dentition has been evaluated by some studies. In the study by Sujitha et al. [28], the most common nonmetric traits in deciduous molars were recorded as the Carabelli’s cusp with 90.6% prevalence, metaconule (the fifth cusp of the second upper molar) with 30.17% and deflecting wrinkle with 87.41%. Díaz et al. [41] concluded that the most common morphological features of the teeth were shoveling, the Carabelli’s cusp, and the sixth cusp. In their study [41], there was a symmetry for morphological features, which was consistent with the present study. Also in the study by Sujitha et al. [42], a significant difference was observed in the mesiodistal width of the first and second maxillary molars between the right and left sides, which was inconsistent with the present study because there was not a significant difference in the present study between the mesiodistal dimensions of the teeth on the right and left sides. Nevertheless, in the present study, two of the teeth showed differences between the buccolingual dimensions of the right and left sides. In their report [42], the mandibular second deciduous molar had the largest mesiodistal dimension and the maxillary second deciduous molar had the largest buccolingual dimension. These results were consistent with the present study. However, the prevalence rates differ in the present study. A study by Avula et al. [29] showed that the right and left teeth were not significantly different and that tooth size was larger in boys compared to girls, which was consistent with the present study.

The strength of the study was the rather broad range of metric and non-metric variables examined. This study was limited by some factors. It would be much better to sample not from orthodontic or pediatric patients, but from a non-patient population. Nevertheless, due to the difficulty of this task, most previous studies were likewise limited to dental populations. Another limitation of this study was the lack of assessment of the role of age; we discarded the individuals’ age data because (1) age has no role in non-metric traits [18], and (2) its very limited effect on metric dental sizes, if any, is extremely small and may appear after many years, probably due to attrition [9, 10] which might be absent in a very short life span of primary dentition, especially given the presence of generalized interdental spacing between the primary teeth. Therefore, age was not considered a variable within this sample of children with primary teeth and thus having very small ranges of age. Although our findings propose objective and clear-cut thresholds for sex identification, these cut-offs should be used with caution because the generalizability of this study is limited to this particular ethnic group, i.e., Iranians –Caucasians originating from Western Asia. Future studies with larger samples and other ethnic groups are needed to verify and possibly adjust the thresholds reported here.

## Conclusions

Within the limitations of this study, it might be concluded that:

1. All the primary molars’ coronal dimensions (both mesiodistal and buccolingual) were useful for sex identification. Especially, the mandibular primary molars were more useful than the maxillary ones. In the mandible, the first primary molar was better than the second molar. The optimum thresholds for sex determination were reported.
2. The prevalences of the nonmetric traits or variations as well as the descriptive statistics for the metric crown widths were reported for the primary molars of Iranian orthodontic and pediatric patients.
3. Sex dimorphism was considerable in buccolingual and mesiodistal crown widths of all the primary molars, but it was rather uncommon in nonmetric traits. It was seen only in the case of deflecting wrinkle on the primary mandibular second molar and tuberculum intermedium (metaconulid) on the primary mandibular second molar, taking into account the prevalences of unilateral and bilateral cases. Without considering the unilateral versus bilateral cases, only deflecting wrinkle on the lower E showed sex dimorphism.
4. The occurrence of nonmetric traits was symmetrical between the right and left sides. All mesiodistal and two buccolingual molar measurements were as well symmetrical; however, two buccolingual measurements were asymmetrical: buccolingual measurements of the maxillary E and mandibular D. Metric measurements were highly correlated between the right and left counterpart teeth.
5. There were three weak-to-moderate correlations between the nonmetric traits of the mandibular second molars.
6. Up to 6 concurrent nonmetric traits were observed in the sample, with 53.6% of the sample showing at least 2 concurrent nonmetric traits at the same time. These concurrent cases were similarly distributed in girls and boys, without any sexual dimorphism.

## Data Availability

The data are available from the corresponding author upon request.
